# Exploration in the mechanism of fucosterol for the treatment of non-small cell lung cancer based on network pharmacology and molecular docking

**DOI:** 10.1038/s41598-021-84380-w

**Published:** 2021-03-01

**Authors:** Xiaoling Li, Baixin Lin, Zhiping Lin, Yucui Ma, Qu Wang, Yushi Zheng, Liao Cui, Hui Luo, Lianxiang Luo

**Affiliations:** 1 Animal Experiment Center of Guangdong Medical University, Zhanjiang, 524023 China; 2 The First Clinical College, Guangdong Medical University, Zhanjiang, 524023 China; 3 The Orthopedic Department, The Affiliated Hospital of Guangdong Medical University, Zhanjiang, 524023 China; 4 Guangdong Key Laboratory for Research and Development of Natural Drugs, Guangdong Medical University, Zhanjiang, 524023 Guangdong China; 5 The Marine Biomedical Research Institute, Guangdong Medical University, Zhanjiang, 524023 China; 6 The Marine Biomedical Research Institute of Guangdong Zhanjiang, Zhanjiang, 524023 Guangdong China; 7 Southern Marine Science and Engineering Guangdong Laboratory (Zhanjiang), Zhanjiang, 524023 Guangdong China

**Keywords:** Cancer, Computational biology and bioinformatics

## Abstract

Fucosterol, a sterol isolated from brown algae, has been demonstrated to have anti-cancer properties. However, the effects and underlying molecular mechanism of fucosterol on non-small cell lung cancer remain to be elucidated. In this study, the corresponding targets of fucosterol were obtained from PharmMapper, and NSCLC related targets were gathered from the GeneCards database, and the candidate targets of fucosterol-treated NSCLC were predicted. The mechanism of fucosterol against NSCLC was identified in DAVID6.8 by enrichment analysis of GO and KEGG, and protein–protein interaction data were collected from STRING database. The hub gene GRB2 was further screened out and verified by molecular docking. Moreover, the relationship of GRB2 expression and immune infiltrates were analyzed by the TIMER database. The results of network pharmacology suggest that fucosterol acts against candidate targets, such as MAPK1, EGFR, GRB2, IGF2, MAPK8, and SRC, which regulate biological processes including negative regulation of the apoptotic process, peptidyl-tyrosine phosphorylation, positive regulation of cell proliferation. The Raf/MEK/ERK signaling pathway initiated by GRB2 showed to be significant in treating NSCLC. In conclusion, our study indicates that fucosterol may suppress NSCLC progression by targeting GRB2 activated the Raf/MEK/ERK signaling pathway, which laying a theoretical foundation for further research and providing scientific support for the development of new drugs.

## Introduction

Lung cancer is the leading cause of cancer death worldwide^1^. Non-small cell lung cancer (NSCLC) accounts for about 85% of lung cancer^2^, and mainly includes lung squamous cell carcinoma(LUSC), lung adenocarcinoma(LUAD)^3^. The principal treatment methods of NSCLC are chemotherapy, surgery, radiotherapy and targeted therapy^4^, but the five-year survival rate as low as 18%, and may lead to serious side effects and drug resistance^5,6^. Therefore, there is an urgent need to develop effective drugs for the treatment of NSCLC.

An estimated 25% of Earth's total species lies in marine species. Many compounds in the ocean have special biological activities and chemical structures recognized as potential medicines for many diseases^7,8^. Most of marine plants extracts have been confirmed to have a variety of bioactivities, including anti-cancer^9,10^, anti-inflammatory^11,12^, anti-viral^13,14^, etc. Marine medicines derived from marine plant extracts have received more and more attention. Fucosterol belongs to algal phytosterol in ethanol extract of brown algae (a kind of macroalgae in the ocean), which has been proved to have multiple biological activities, including antioxidant^15–17^, anti-inflammatory^18–20^, anticancer^21^, antimicrobial^22^, anti-depression^23^, etc. Previous researches have reported fucosterol on anti-cervical cancer^21^, anti-leukemia^24^, anti-colorectal cancer^25^, etc., but there are few studies on the mechanism of fucosterol in treating NSCLC about its potential therapeutic targets and related pathways in detail.

In the past few years, network pharmacology, as a new discipline based on system biology and multi-direction pharmacology, can effectively implement the predictive analysis of drug action mechanisms^26^, identify new drug targets^27^, and better explain the mechanism of interactions between bioactive molecules and cellular pathways. Molecular docking technology is an advantageous tool for modernization research, which can achieve virtual screening of drugs^28^. Reverse molecular docking refers to the docking of a small molecule drug in a group of potential binding cavities of clinically relevant large molecule targets. Through detailed analysis of its binding characteristics, the potential small molecule targets are identified and ranked according to the binding tightness^29,30^.

**Figure 1:**
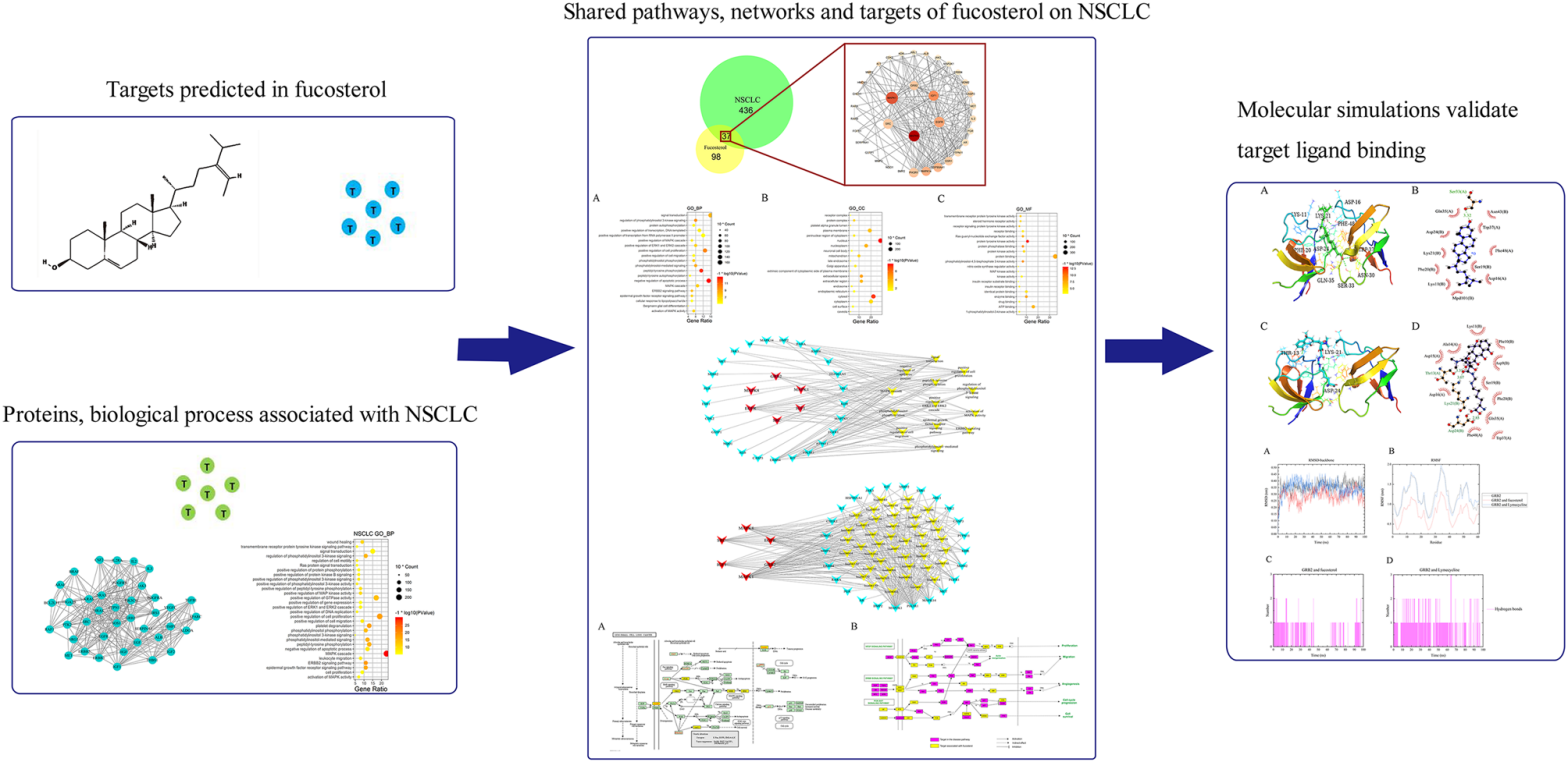
A stepwise workflow showed the mechanism of fucosterol against NSCLC through network pharmacology.

In this study, we employed network pharmacology and molecular docking to explore the effects and mechanisms of fucosterol on NSCLC. A detailed description of the procedure is shown in Fig. 1. We aimed to provide a new idea for the treatment of NSCLC and promote the development of new drugs.

## Materials and methods

### Data preparation

#### Acquisition of fucosterol chemical structure

The PubChem database (https://pubchem.ncbi.nlm.nih.gov/) was used to retrieve the 3D chemical structure of fucosterol, saved it as “sdf” format, and converted to the mol2 format by Pymol2.4 for minimizing the MM2 energy.

#### Prediction and screening of fucosterol targets

Fucosterol's mol2 format was uploaded to the PharmMappper database (http://www.lilab-ecust.cn/pharmmapper/) to determine the fucosterol-related predictive targets^31–33^. Set parameters: Generate Conformers: Yes; Maximum Generated Conformations: 300; Select Target Set: Human Protein Target Only; Number of Reserved Matched Targets: 300. The results of predictive targets with z-score > 0 as screening criteria were retained for further analysis.

#### Protein and gene information correction

Fucosterol-related predictive targets were input into UniProtKB database (http://www.uniprot.org/) to collect the official symbols with the species limited to “*Homo sapiens*” by eliminating duplicate and nonstandard targets.

#### Identification of fucosterol and NSCLC common targets

NSCLC-related targets were gathered from GeneCards database (https://www.genecards.org/) using the phrase “non-small cell lung cancer” as a keyword, and the top 500 targets were retained according to the relevance score. The predictive targets of fucosterol were compared with the NSCLC-related targets in BioVenn (http://www.biovenn.nl/index.php) to identify the candidate targets of fucosterol for NSCLC treatment.

#### Collection of protein–protein interaction data

The data on protein–protein interaction (PPI) were obtained from STRING database 11.0 (https://string-db.org/) with uploading the candidate targets while the species limited to “*Homo sapiens*” and the confidence score > 0.700 (low confidence: score < 0.400; medium confidence: 0.400 < score < 0.700; high confidence: 0.700 < score < 0.900; highest confidence: score > 0.900).

#### Gene ontology and KEGG analysis

DAVID 6.8 (https://david.ncifcrf.gov/home.jsp) was applied to carry out GO enrichment analysis and KEGG pathway enrichment analysis of the candidate targets. First, the species option was selected as “*Homo sapiens*”. Second, four analysis items were selected: KEGG pathway, cellular components, molecular functions, and biological processes. Third, enriched GO terms and KEGG pathways were screened for *P*-value ≤ 0.05 using the Bonferroni correction. Finally, based on the third step, KEGG pathway and the top 20 items of GO with ranking by their *P*-value were chosen for further analysis.

### Network construction

#### Network construction method

The Cytoscape3.7.2 software (https://cytoscape.org/) was utilized for network topology analysis and network visualization. Construct the following networks: (1) Cluster 1 for NSCLC network; (2) Fucosterol-NSCLC PPI network; (3) Important biological processes and the candidate targets crosstalk network; (4) Target-pathway interaction network.

#### Cluster for NSCLC network

The disease module is a set of network components that collectively disrupts cellular function and lead to a specific disease phenotype. Owing to the disease module, the functional module and the topology module have the same meaning in the network, the function module is equal to the topology module, and the disease can be considered as interference and destruction of functional module^34,35^. The plug-in MCODE of Cytoscape3.7.2 software was used to analyze the protein interaction network of NSCLC to identify the dense regions, and the first cluster of results was retained.

#### Screening of important biological processes

The biological processes of the candidate targets retained based on the mentioned steps were compared with the results obtained by the cluster 1 network enrichment analysis of NSCLC. Then, the interaction network was established to show the associations between the important biological processes and the candidate targets.

#### Construction of target-pathway network

Of the enriched KEGG pathways with a *P*-value of < 0.05, the disease and generalized pathways were removed, and visualized the relationship with candidate targets by the target-pathway interaction network.

#### NSCLC-related pathways map construction

For exploring the comprehensive mechanism of fucosterol treatment of NSCLC, an integrated pathway map related to NSCLC was established, including the NSCLC disease pathway map and the key signal transduction pathways map.

#### Prediction of binding between fucosterol and GRB2

In this work, the initial structure of the ligand-free structure of the GRB2 (PDB code: 6SDF), was obtained from X-ray diffraction in Protein Data Bank, and the 3D structure of fucosterol (compound CID: 5281328) and Lymecycline (compound CID: 54707177) were obtained from Pubchem. Lymecycline was used as a positive control. Then, we used AutoDock 4 which based on the standard docking procedure for a rigid protein and a flexible ligand. At first, we used pymol2.4 to remove the water molecules and the original ligands from the GRB2, and then used AutoDock4 to add hydrogen and used the vina to dock the GRB2 with fucosterol and Lymecycline, and then selected the compound with the best binding effect. Finally, we used pymol2.4 and ligplot2.3 to observe and analyze the interaction and binding mode between ligand and receptor.

#### Molecular dynamics simulations

Lymecycline and fucosterol with GRB2 were rationally docked in our previous work. This complex was analyzed by all atom molecular dynamics (MD) simulations for 50 ns. All the simulations were carried out with Gromacs 2020.2 software package and gromos54a7_atb.ff force field. The force field parameters of fucosterol and Lymecycline was generated by ATB (https://atb.uq.edu.au/register.py). To ensure the total charge neutrality of the simulated system, the corresponding amount of sodium ions were added to the system to replace water molecules to produce solvent boxes of the appropriate size. Initial the energy of 50,000 steps of the whole system was minimized by (EM) at 300 K. Subsequently, the systems were equilibrated by position restraint simulations of NVT and NPT ensembles. Equilibrated systems were used to simulate a 50 ns no restraint production run. In the end MD analyses were performed, this includes root mean square deviation (RMSD), root mean square fluctuations (RMSF) and Hydrogen bond analysis. The data of RMSD, RMSF and Hydrogen bond analysis is generated by Origin2019b (https://www.originlab.com/).

#### GRB2 expression and immune infiltrates

In order to explore the correlation between GRB2 expression and immune infiltrates in LUAD, the TIMER2.0 database (http://timer.cistrome.org), a friendly comprehensive tool set for systematical analysis of immune infiltrates across diverse cancer types^36–38^, was carried out. There were many immune cells in the TIMER database. We chose CD4 + T cells, CD8 + T cells, B cells, neutrophils, macrophages, myeloid dendritic cells to conduct a new analysis. Besides, we performed the association between gene GRB2 and EGFR after adjusting tumor purity in the correction section in TIMER2.0 database.

#### Prognostic values of GRB2

The correlation between GRB2 expression and survival in lung adenocarcinoma was analyzed by Kaplan–Meier plotter (http://kmplot.com/analysis/), a tool for assessing the functions of 54,675 genes and 10,188 tumor tissue samples, including breast cancer, ovarian cancer, lung cancer, and gastric cancer.

## Results

### Predicted target screening of fucosterol

A total 210 predictive targets were identified from the PharmMapper database, and ultimately, 135 official symbols of fucosterol related targets for limiting to “*Homo sapiens*” were obtained in the UniProtKB database after filtering by z-score > 0 (Table 1).

**Table 1:** Prediction targets of fucosterol.

Pharma model	Gene symbol	z-score
3f0r	HDAC8	2.82743
1qyx	HSD17B1	2.70536
1o6u	SEC14L2	2.68742
1n83	RORA	2.39228
1a28	PGR	2.2175
1ya3	NR3C2	2.13319
1s9j	MAP2K1	2.12046
1q22	SULT2B1	1.98504
1pic	PIK3R1	1.88125
1lv2	HNF4G	1.83771
1rhr	CASP3	1.80882
1osh	NR1H4	1.79715
830c	MMP13	1.78365
1rbp	RBP4	1.71609
1lho	SHBG	1.70322
1mrq	AKR1C1	1.70301
1sz7	TRAPPC3	1.6669
3czr	HSD11B1	1.65102
2piw	AR	1.6069
1fd0	RARG	1.59522
1d4p	F2	1.55496
1gse	GSTA1	1.54788
1uhl	NR1H3	1.52908
1hrk	FECH	1.52472
2iiv	DPP4	1.50916
1s0z	VDR	1.49636
1dkf	RARA	1.49513
2i6b	ADK	1.38413
1o1v	FABP6	1.35079
1ydt	PRKACA	1.31178
2shp	PTPN11	1.28799
2fjm	PTPN1	1.27676
2j14	PPARD	1.27187
1 × 0n	GRB2	1.2585
3bbt	ERBB4	1.25573
1l8j	PROCR	1.23987
2vx0	EPHB4	1.23974
1he3	BLVRB	1.19363
1pq2	CYP2C8	1.19205
1j99	SULT2A1	1.08311
1cbs	CRABP2	1.06101
1sm2	ITK	1.04514
1upw	NR1H2	1.03078
1qpd	LCK	1.00159
1uym	HSP90AB1	0.997933
1hov	MMP2	0.978531
1h9u	RXRB	0.976261
1u59	ZAP70	0.97084
1fe3	FABP7	0.963876
19gs	GSTP1	0.95545
2hzi	ABL1	0.954306
1yvj	JAK3	0.922657
1m48	IL2	0.917557
1t84	WAS	0.909365
1dxo	NQO1	0.886825
1j78	GC	0.867991
2p4i	TEK	0.803103
1iz2	SERPINA1	0.79593
1oiz	TTPA	0.795317
1ln3	PCTP	0.794336
1t4e	MDM2	0.78729
1xap	RARB	0.775562
1qkt	ESR1	0.767258
1r9o	CYP2C9	0.742597
1qip	GLO1	0.733894
1hmt	FABP3	0.731703
1bl6	MAPK14	0.730803
2rfn	MET	0.721237
1i7g	PPARA	0.707586
1pmn	MAPK10	0.678619
1e7a	ALB	0.644744
2oi0	ADAM17	0.634049
1cg6	MTAP	0.623444
1ma0	ADH5	0.608206
1g3m	SULT1E1	0.594411
1xjd	PRKCQ	0.557908
2uwd	HSP90AA1	0.555214
1oj9	MAOB	0.540688
1njs	GART	0.529254
1xvp	NR1I3	0.502645
2pjl	ESRRA	0.488399
1nhz	NR3C1	0.486782
1j96	AKR1C2	0.454473
2ipw	AKR1B1	0.434142
1p49	STS	0.426598
1xbb	SYK	0.421996
1okl	CA2	0.418201
2qu2	BACE1	0.398063
1ih0	TNNC1	0.394868
1sa4	FNTA	0.387846
1l6l	APOA2	0.385162
2fgi	FGFR1	0.36325
2pe0	PDPK1	0.355588
2h8h	SRC	0.34725
2itp	EGFR	0.342717
1xor	PDE4D	0.333997
1jqe	HNMT	0.312737
1shj	CASP7	0.309471
2iku	REN	0.285831
2pg2	KIF11	0.27567
3bgp	PIM1	0.259029
2ywp	CHEK1	0.245709
1t46	KIT	0.241973
1s95	PPP5C	0.227167
1itu	DPEP1	0.226319
2b53	CDK2	0.222033
2jbp	MAPKAPK2	0.216487
3cjg	KDR	0.212231
1n7i	PNMT	0.205326
1egc	ACADM	0.198463
1so2	PDE3B	0.191704
1l9n	TGM3	0.19101
2bxr	MAOA	0.187567
1hw8	HMGCR	0.158181
1tt6	TTR	0.155605
2o9i	NR1I2	0.148658
1 × 89	LCN2	0.147746
1j4i	FKBP1A	0.145396
1xlz	PDE4B	0.141599
1s8c	HMOX1	0.137498
1tjj	GM2A	0.121157
1mx1	CES1	0.0987688
2g01	MAPK8	0.0924675
1reu	BMP2	0.0907675
1fzv	PGF	0.0881642
1s1p	AKR1C3	0.0878495
1r7y	ABO	0.0714749
1pme	MAPK1	0.0689871
1g4k	MMP3	0.0676583
1h6g	CTNNA1	0.0673048
1vj5	EPHX2	0.0595376
2nn7	CA1	0.049744
2pin	THRB	0.0116825
1gzr	IGF1	0.00629983

### Cluster analysis for NSCLC-related targets

**Figure 2:**
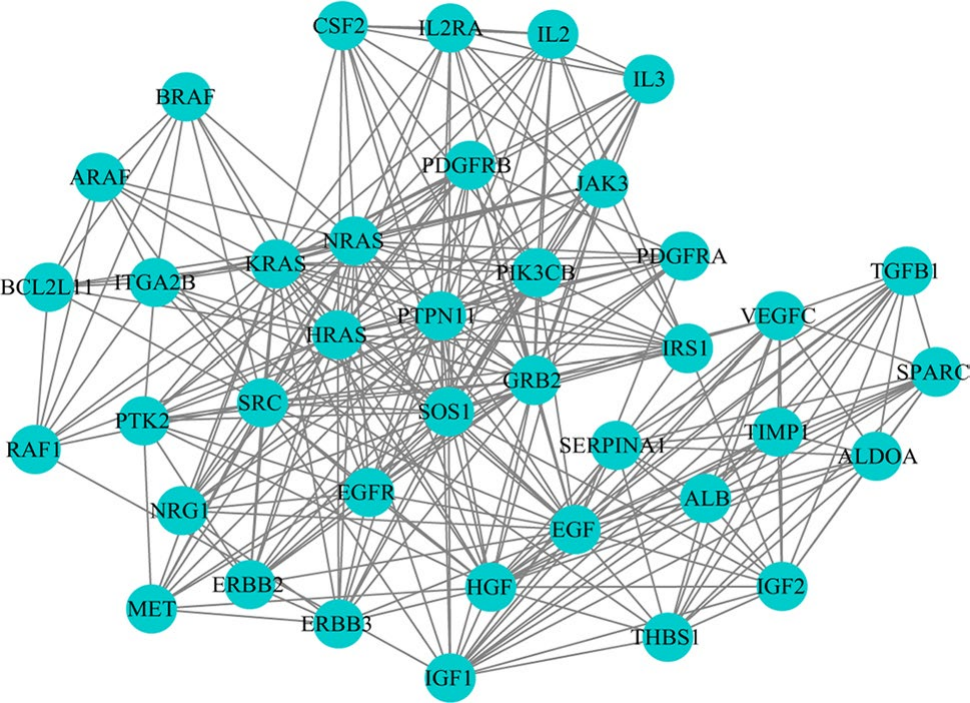
Cluster 1 for NSCLC network in Cytoscape3.7.2 (https://cytoscape.org/).

**Figure 3:**
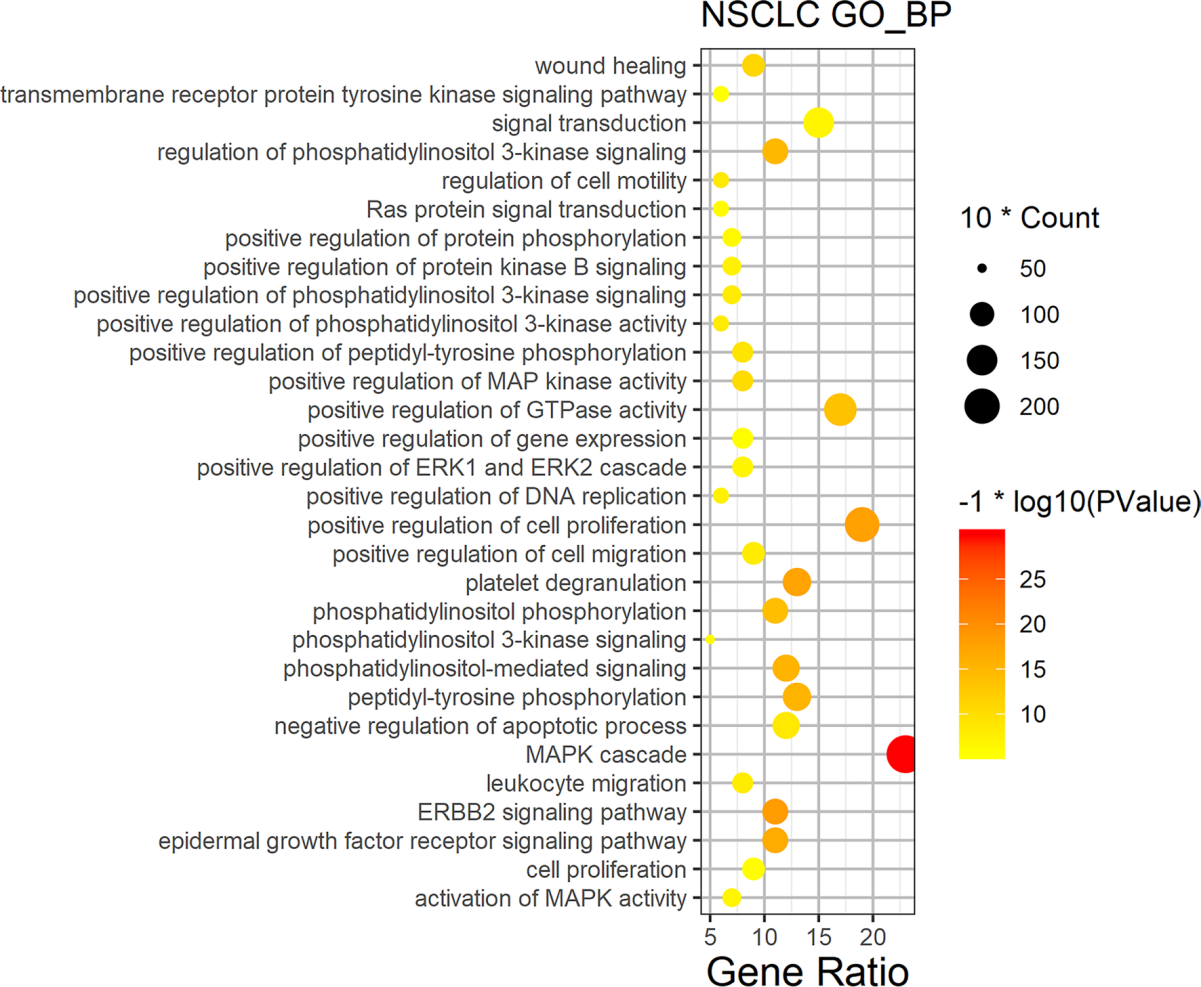
Biological process bubble diagram of cluster 1. (The color scales indicate different thresholds of adjusted *p*-value, and the sizes of the dots represent the gene count of each term.)

Although we selected high-relevance score of NSCLC targets from GeneCards, the NSCLC bio-network was still huge. To further analyze biological processes of the function module in NSCLC network, we obtained protein–protein interaction network (PPI) data for NSCLC, and clustered to find out the topology module of NSCLC’s PPI network. The cluster 1 was retained since it is the most significant for PPI network of NSCLC (Fig. 2), and the genes of cluster 1 was shown in Table1. Functional enrichment analyses of GO biological process on the genes of cluster 1 were performed by DAVID6.8, showing cell proliferation, apoptosis, cell cycle, angiogenesis, NSCLC gene expression, invasion and migration, signal transduction, and NSCLC related signaling pathways (Fig. 3).

### Analysis of fucosterol-NSCLC PPI network

**Figure 4:**
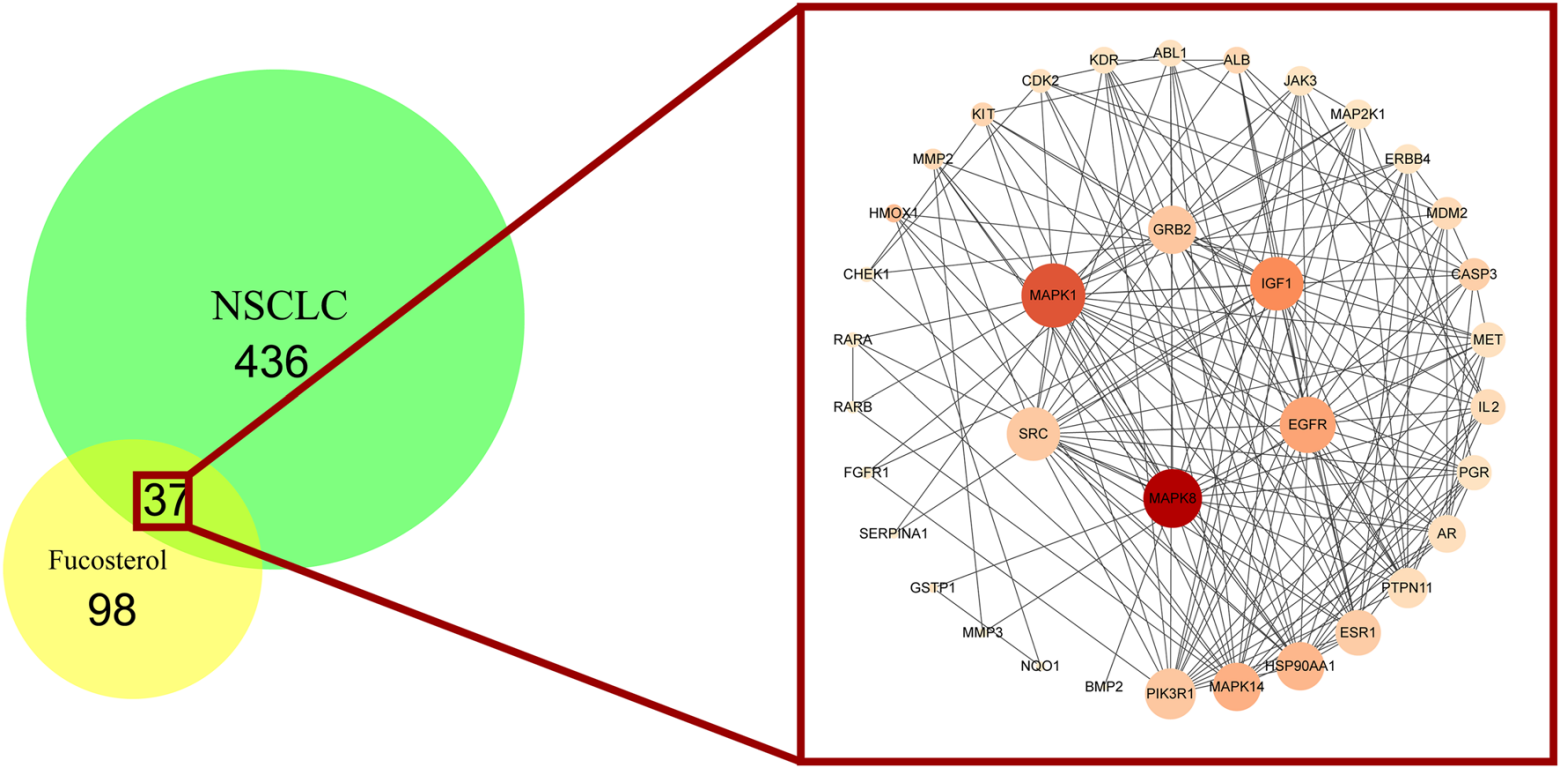
The PPI network constructed for the candidate targets of fucosterol treated NSCLC using Cytoscape3.7.2 (https://cytoscape.org/).

In order to construct the interaction network between proteins and dig out the core regulatory genes, the fucosterol-NSCLC PPI network of the candidate targets was performed (Fig. 4). Fucosterol shared 37 targets with NSCLC, and the network consisted of 36 nodes (one of the target proteins does not interact with others) and 177 edges. The color of each node is related to its degree; the darker nodes have the larger value of Degree. The size of the node is linked to its Edge; the bigger nodes have the larger value of Edge Betweenness. Based on the network topology analysis, the betweenness centrality is 0.0261, the average node degree is 9.83, the average closeness centrality is 0.535, which suggests the presence of a central hub between candidate targets. Six hub genes were extracted according to betweenness centrality, node degree and closeness centrality. The hub genes were speculated to play a significant role in fucosterol treated NSCLC, including EGFR, MAPK8, MAPK1, GRB2, SRC, IGF1 (Table 2).

**Table 2:** Hub genes in NSCLC treated fucosterol.

Name	Betweenness centrality	Closeness centrality	Degree
GRB2	0.03441407	0.614035	16
EGFR	0.06936033	0.648148	19
MAPK1	0.12911792	0.729167	22
SRC	0.03183796	0.660377	18
IGF1	0.09323304	0.660377	18
MAPK8	0.18473443	0.7	20

### Gene ontology enrichment analysis for candidate targets

**Figure 5:**
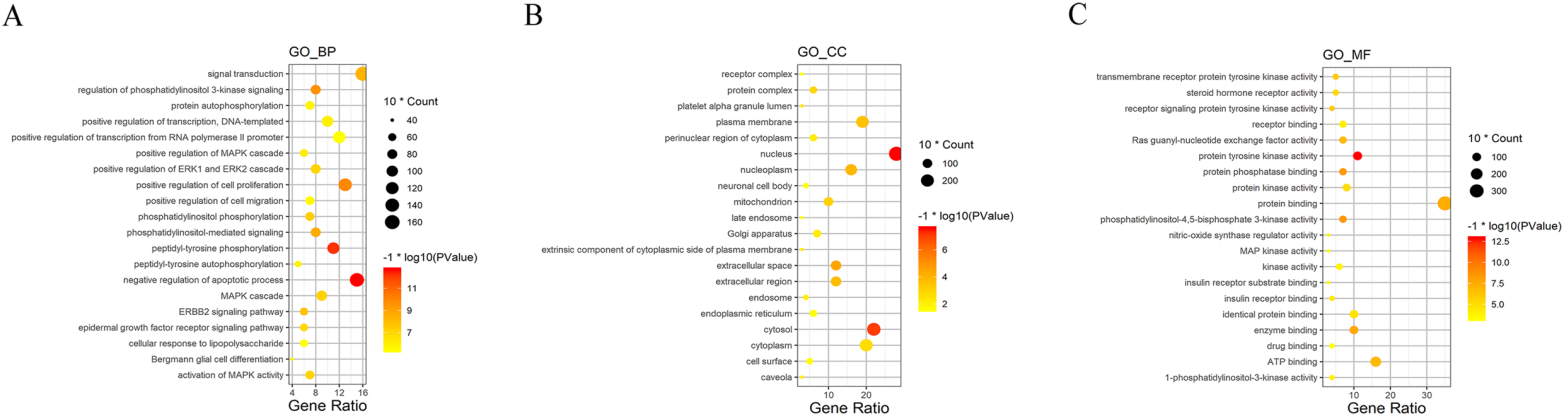
GO analysis of candidate targets. GO enrichment analysis identified genes involved in (**A**) biological processes, (**B**) cellular components, and (**C**) molecular functions.

**Figure 6:**
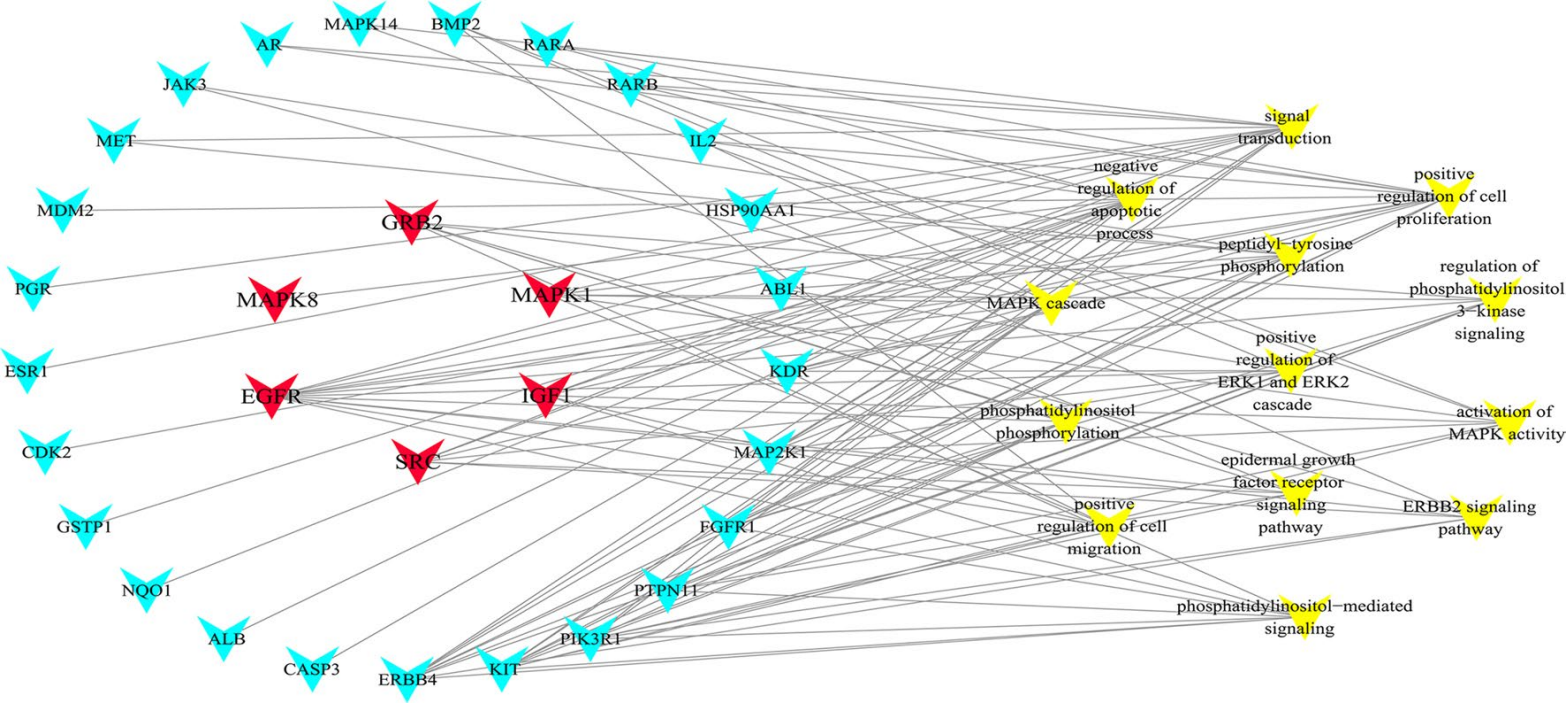
The network made in Cytoscape3.7.2 (https://cytoscape.org/) depicted the relationship of candidate targets and important biological processes of fucosterol treated NSCLC. (Node in red is the hub gene, node in blue is the candidate targets, and yellow is the specific biological processes.)

GO enrichment yields a deeper understanding on the gene function and biological significance of the candidate targets of fucosterol treated NSCLC on a systematic level. To obtain the biological processes, molecular functions, and cellular components of the candidate targets, we performed a GO enrichment analysis, and displayed the top 20 significantly terms (*p*-value ≤ 0.05) of each module in Fig. 5. It is suggested that the candidate targets could act through protein tyrosine kinase activity, protein phosphatase binding, negative regulation of apoptotic process, peptidyl-tyrosine phosphorylation, positive regulation of cell proliferation in the nucleus, cytosol, extracellular space, nucleoplasm, extracellular region. Among them, 14 vital biological processes directly affect cluster 1 of NSCLC disease module were presented independently by connecting with the candidate targets (Fig. 6). This network diagram revealed candidate targets were mainly involved in cell proliferation and apoptosis, angiogenesis, cell migration and signal transduction, and illustrated that hub genes were strongly associated with various biological processes.

### Evaluation of target-pathway network

**Figure 7:**
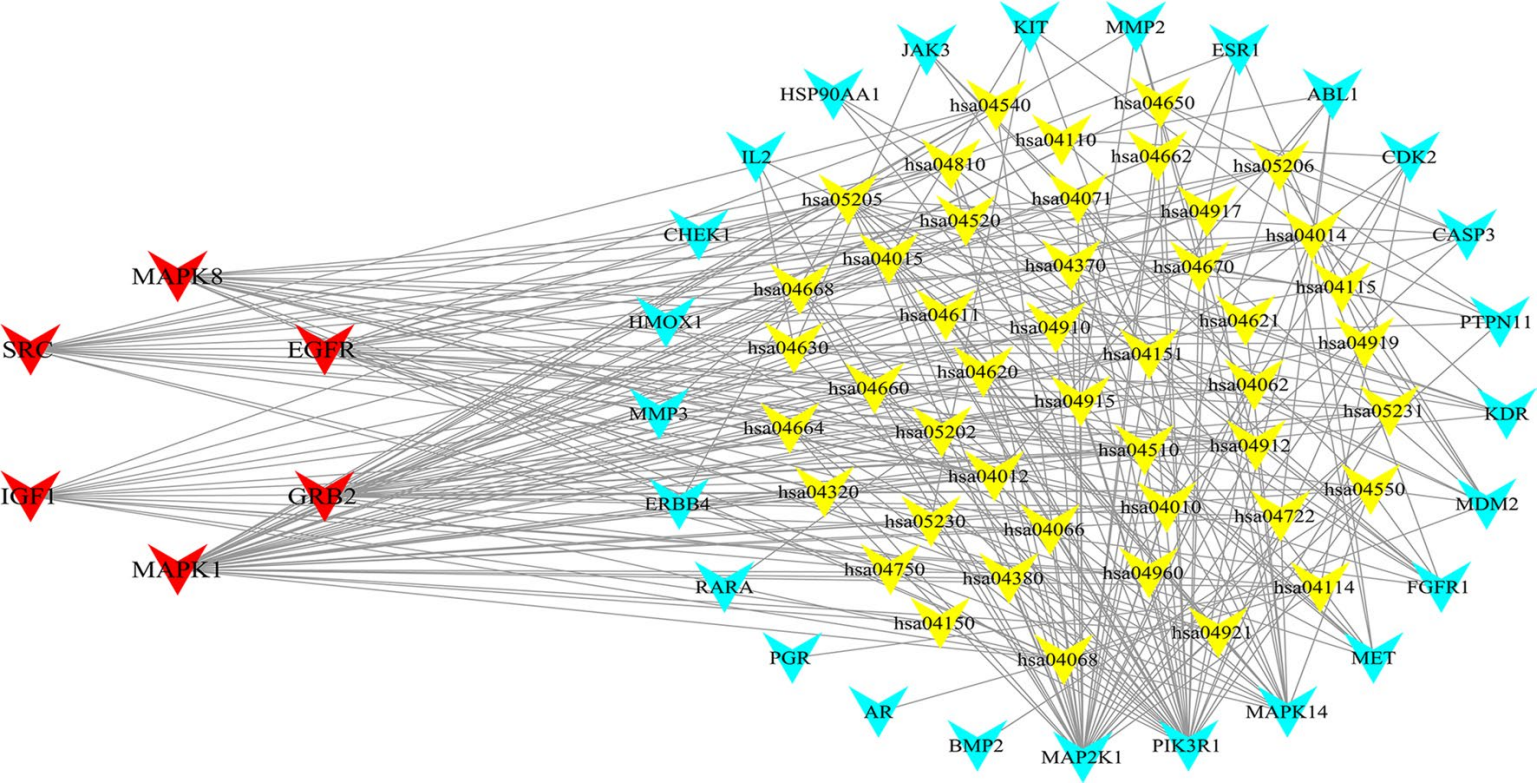
The Target-pathway network, constructed in Cytoscape3.7.2 (https://cytoscape.org/), was built by the candidate targets and a pathway if the pathway was lighted at the target. (Yellow nodes represent signaling pathway from enrichment analysis. Blue nodes represent the candidate targets in fucosterol. Red nodes represent the hub genes screened by PPI network.)

To explain better the mechanisms of fucosterol treatment of NSCLC at the pathway level, 73 pathways were obtained by mapping 37 candidate targets (Table 3). After getting rid of generalized and other disease terms, a total of 45 pathways and 37 candidate targets constituted the target-pathway interaction network (Fig. 7). Some targets are mapped to multiple pathways and multiple targets also regulated various pathways suggesting that candidate targets may mediate interaction and crosstalk of different pathways. The pathways may be the major factor for fucosterol's resistance to NSCLC, that is, PI3K-Akt signaling pathway, VEGF signaling pathway, ErbB signaling pathway. The PI3K-Akt signaling pathway is widely recognized as a prominent cancer signaling pathway, and is closely related to affect the proliferation, survival and apoptosis of NSCLC cells^39–41^. Also, VEGF signaling pathway involves in tumor cell-dependent continuous vascular supply and thus has a profound effect on tumor cell growth and metastasis^42^. In addition, owing to interaction of ErbB receptors with many signal transduction molecules, which can activate multiple intracellular pathways, the ErbB signaling pathway plays a significant role in the development of cancer^43^. As mentioned, the three pathways are closely relevant to NSCLC treatment. Besides, it has been proved that fucosterol exerts antiproliferative effects to achieve the therapeutic purpose of lung cancer through targeting Raf/MEK/ERK signaling pathway^44^. And, according to the KEGG map of the three pathways (hsa04151: PI3K-Akt signaling pathway, hsa04012: ErbB signaling pathway, hsa04370: VEGF signaling pathway), all of them can activate the downstream pathway–Raf/MEK/ERK signaling pathway. Therefore, the three signal transduction pathways were selected as key signaling pathways for fucosterol in the treatment of NSCLC for integration and further analysis.

**Table 3:** KEGG analysis of candidate targets of fucosterol for NSCLC.

Term	Pathways	Count	*p*-value
hsa05200	Pathways in cancer	21	1.21E−16
hsa05205	Proteoglycans in cancer	17	3.47E−16
hsa04151	PI3K-Akt signaling pathway	15	4.36E−10
hsa04014	Ras signaling pathway	13	5.06E−10
hsa05215	Prostate cancer	11	9.84E−12
hsa04015	Rap1 signaling pathway	11	5.35E−08
hsa04068	FoxO signaling pathway	10	1.42E−08
hsa04510	Focal adhesion	10	5.84E−07
hsa04914	Progesterone-mediated oocyte maturation	9	8.41E−09
hsa04012	ErbB signaling pathway	9	8.41E−09
hsa04915	Estrogen signaling pathway	9	2.36E−08
hsa04550	Signaling pathways regulating pluripotency of stem cells	9	3.57E−07
hsa05203	Viral carcinogenesis	9	6.39E−06
hsa05218	Melanoma	8	4.93E−08
hsa04917	Prolactin signaling pathway	8	4.93E−08
hsa04912	GnRH signaling pathway	8	2.79E−07
hsa04722	Neurotrophin signaling pathway	8	1.85E−06
hsa05161	Hepatitis B	8	6.56E−06
hsa04010	MAPK signaling pathway	8	2.33E−04
hsa05206	MicroRNAs in cancer	8	4.92E−04
hsa05230	Central carbon metabolism in cancer	7	6.71E−07
hsa05214	Glioma	7	7.37E−07
hsa05120	Epithelial cell signaling in Helicobacter pylori infection	7	8.84E−07
hsa05220	Chronic myeloid leukemia	7	1.36E−06
hsa04668	TNF signaling pathway	7	1.38E−05
hsa05219	Bladder cancer	6	1.66E−06
hsa05223	Non-small cell lung cancer	6	8.03E−06
hsa05221	Acute myeloid leukemia	6	8.03E−06
hsa04370	VEGF signaling pathway	6	1.23E−05
hsa05211	Renal cell carcinoma	6	1.81E−05
hsa04664	Fc epsilon RI signaling pathway	6	2.10E−05
hsa04066	HIF-1 signaling pathway	6	1.11E−04
hsa04660	T cell receptor signaling pathway	6	1.35E−04
hsa05231	Choline metabolism in cancer	6	1.41E−04
hsa04114	Oocyte meiosis	6	2.21E−04
hsa04919	Thyroid hormone signaling pathway	6	2.60E−04
hsa04650	Natural killer cell mediated cytotoxicity	6	3.43E−04
hsa05169	Epstein-Barr virus infection	6	3.43E−04
hsa04380	Osteoclast differentiation	6	4.76E−04
hsa05160	Hepatitis C	6	5.10E−04
hsa04062	Chemokine signaling pathway	6	2.30E−03
hsa04810	Regulation of actin cytoskeleton	6	3.89E−03
hsa05213	Endometrial cancer	5	1.28E−04
hsa05210	Colorectal cancer	5	2.54E−04
hsa05131	Shigellosis	5	2.87E−04
hsa05212	Pancreatic cancer	5	3.05E−04
hsa04115	p53 signaling pathway	5	3.43E−04
hsa04520	Adherens junction	5	4.29E−04
hsa04540	Gap junction	5	9.67E−04
hsa04750	Inflammatory mediator regulation of TRP channels	5	1.45E−03
hsa05142	Chagas disease (American trypanosomiasis)	5	1.80E−03
hsa04620	Toll-like receptor signaling pathway	5	1.93E−03
hsa04071	Sphingolipid signaling pathway	5	3.04E−03
hsa04910	Insulin signaling pathway	5	5.01E−03
hsa04630	Jak-STAT signaling pathway	5	5.97E−03
hsa05202	Transcriptional misregulation in cancer	5	9.76E−03
hsa05164	Influenza A	5	1.12E−02
hsa05152	Tuberculosis	5	1.19E−02
hsa04320	Dorso-ventral axis formation	4	3.25E−04
hsa04621	NOD-like receptor signaling pathway	4	2.78E−03
hsa04662	B cell receptor signaling pathway	4	5.03E−03
hsa05133	Pertussis	4	6.34E−03
hsa05145	Toxoplasmosis	4	1.80E−02
hsa04670	Leukocyte transendothelial migration	4	2.02E−02
hsa04110	Cell cycle	4	2.46E−02
hsa04611	Platelet activation	4	2.78E−02
hsa05162	Measles	4	2.95E−02
hsa04921	Oxytocin signaling pathway	4	4.00E−02
hsa04960	Aldosterone-regulated sodium reabsorption	3	1.66E−02
hsa04930	Type II diabetes mellitus	3	2.45E−02
hsa04150	mTOR signaling pathway	3	3.48E−02
hsa04730	Long-term depression	3	3.70E−02

### Analysis of the NSCLC-related pathways map

**Figure 8:**
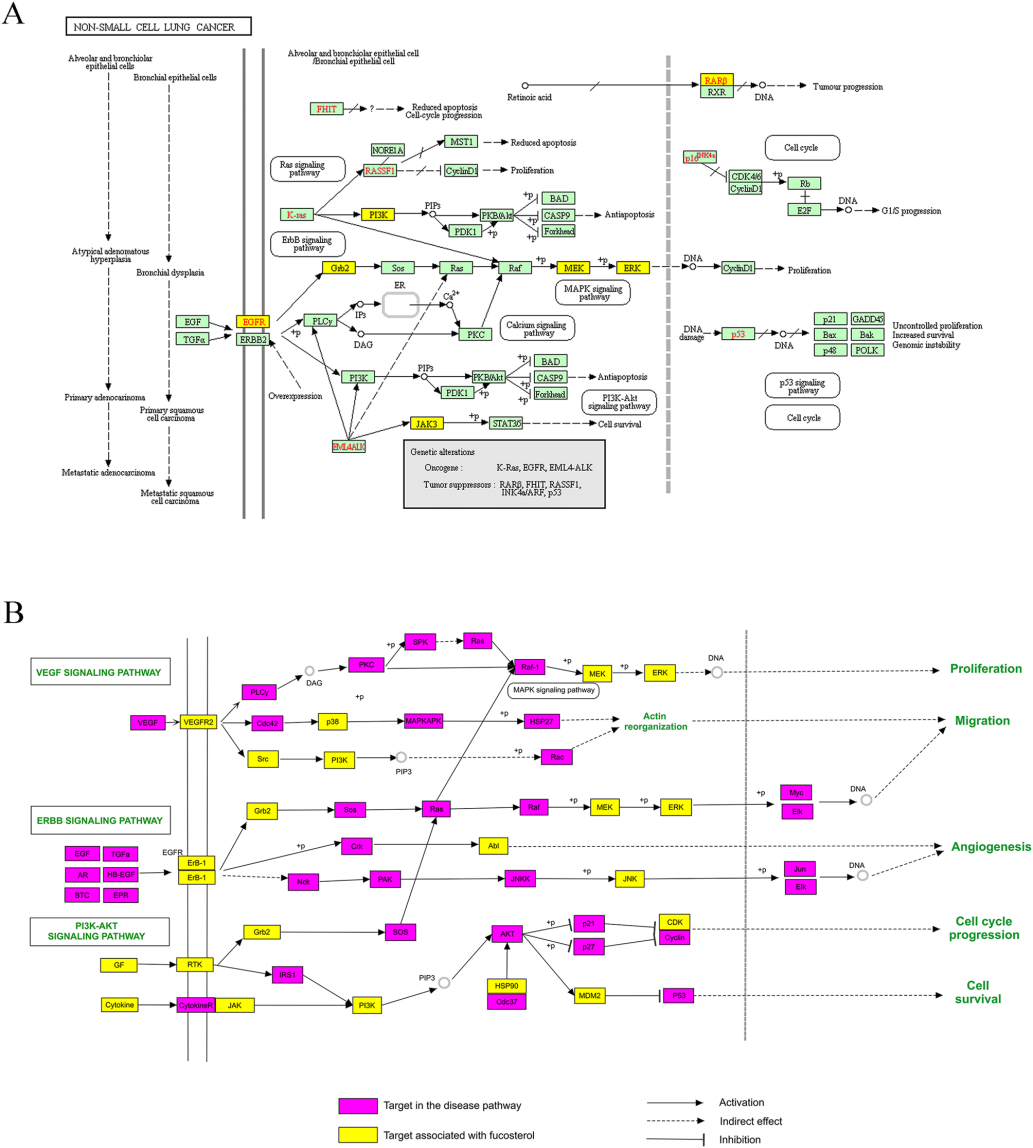
The integrated pathways map of fucosterol in the treatment of NSCLC include (**A**) the non-small cell lung cancer pathway, marked in yellow as the functional targets of fucosterol, and (**B**) the key signal transduction pathways.

To intuitively explicate the complex mechanism of fucosterol treated NSCLC, an integrated pathway map (Fig. 8) was constructed by integrating the NSCLC disease pathway map and the key signal transduction pathways map that obtained from evaluating of target-pathway network. The key signal transduction pathways map comprises of three signaling pathways: hsa04151: PI3K-Akt signaling pathway, hsa04012: ErbB signaling pathway, hsa04370: VEGF signaling pathway. As shown in Fig. 8, the signaling transduction pathways and NSCLC disease pathway reflect multiple modules such as cell proliferation, apoptosis, migration and angiogenesis. From the perspective of NSCLC disease pathway map (Fig. 8A), fucosterol treatment of NSCLC might enable crosstalk of the inflammatory module and the tumor module, involving the joint action of multiple pathways. According to the candidate targets shown in the Fig. 8A, fucosterol may be able to achieve the purpose of treating NSCLC through activating the Ras signaling pathway, PI3K-Akt signaling pathway, ErbB signaling pathway and MAPK signaling pathway to regulate cell proliferation and apoptosis. From the perspective of the key signal transduction pathways(Fig. 8B), both VEGF signaling pathway and ErbB signaling pathway can activate downstream signals to control angiogenesis, affect cell proliferation and migration, and play a crucial role in the development and metastasis of tumors^45–47^. Meanwhile, the PI3K-Akt signaling pathway participates in the regulation of cell cycle progression^48–50^. According to this map, the three signaling pathways can jointly activate the Raf/MEK/ERK signaling pathway reported to play an anti-proliferation role in curing cancer^44,51,52^. More importantly, from the observation in this map, GRB2 can activate the downstream pathways–Raf/MEK/ERK signaling pathway through ErbB signaling pathway and PI3K-Akt signaling pathway, which may make GRB2 one of the potential therapeutic targets for fucosterol in the treatment of NSCLC. Moreover, GRB2 was presumed to be one of the six hub genes of fucosterol therapy for NSCLC base on fucosterol-NSCLC PPI network topology analysis. Collectively, GRB2 is speculated to play an important role in fucosterol treated NSCLC. We will further discuss GRB2 targeted by fucosterol for treating NSCLC.

### Molecular docking validation of GRB2

**Figure 9:**
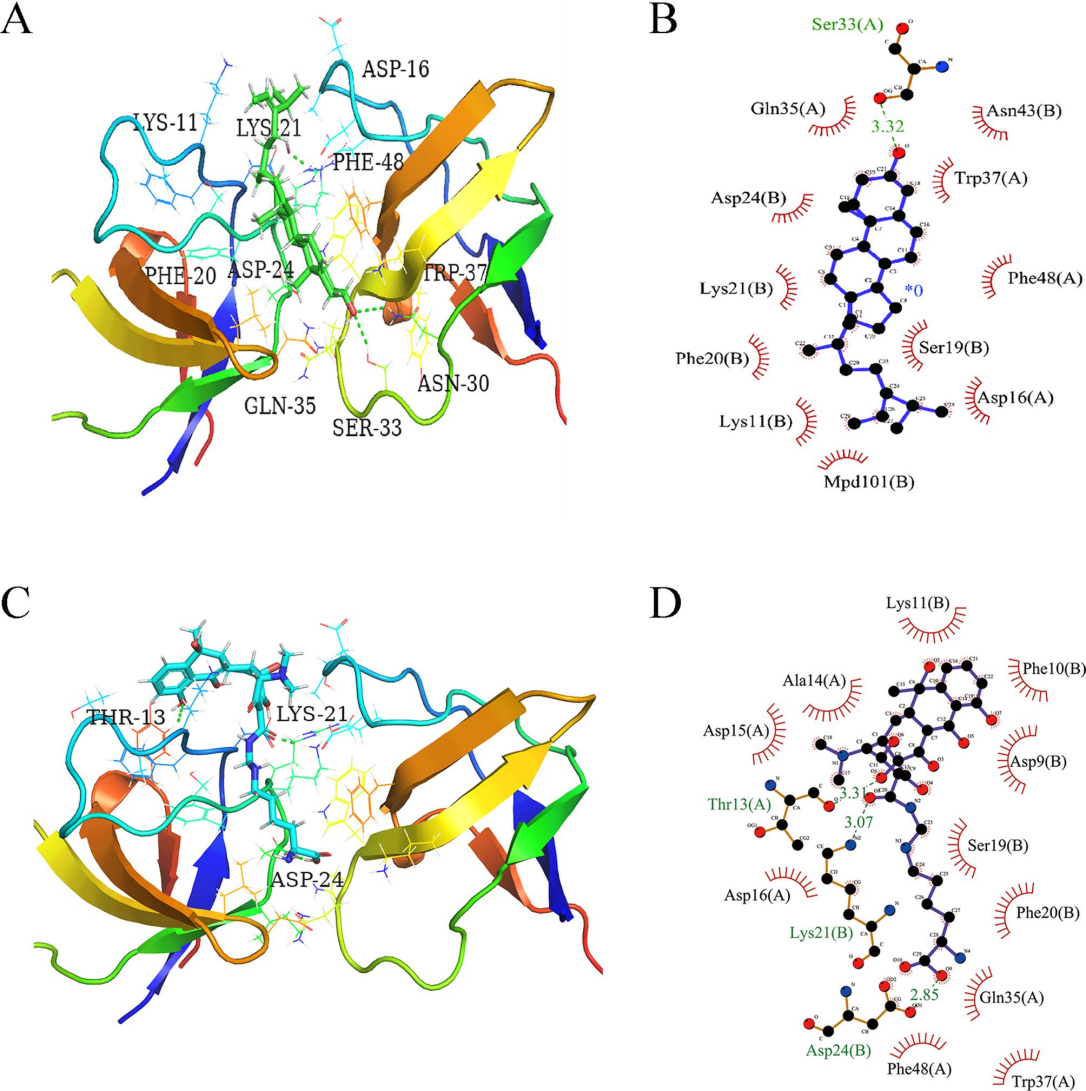
Fucosterol and Lymecycline showing molecular interactions with GRB2 and 2D representation of H-bonds and hydrophobic interactions of fucosterol and Lymecycline with GRB2. (**A**,**B**) Fucosterol and GRB2. (**C,D**) Lymecycline and GRB2. The green stick is shown as fucosterol, the blue stick is shown as lymecycline, the hydrogen bond is shown as the green dotted line. Ligand is colored and represented in purple color, hydrogen bonda is displayed in green dotted lines, red stellations represent hydrophobic interactions, and bonds of proteins are shown in brown color. Figure (**A**&**C**) were created from Pymol2.4 (https://pymol.org), (**B**&**D**) were made in Ligplot2.3 (http://www.csb.yale.edu/userguides/graphics/ligplot/manual/index.html).

**Figure 10:**
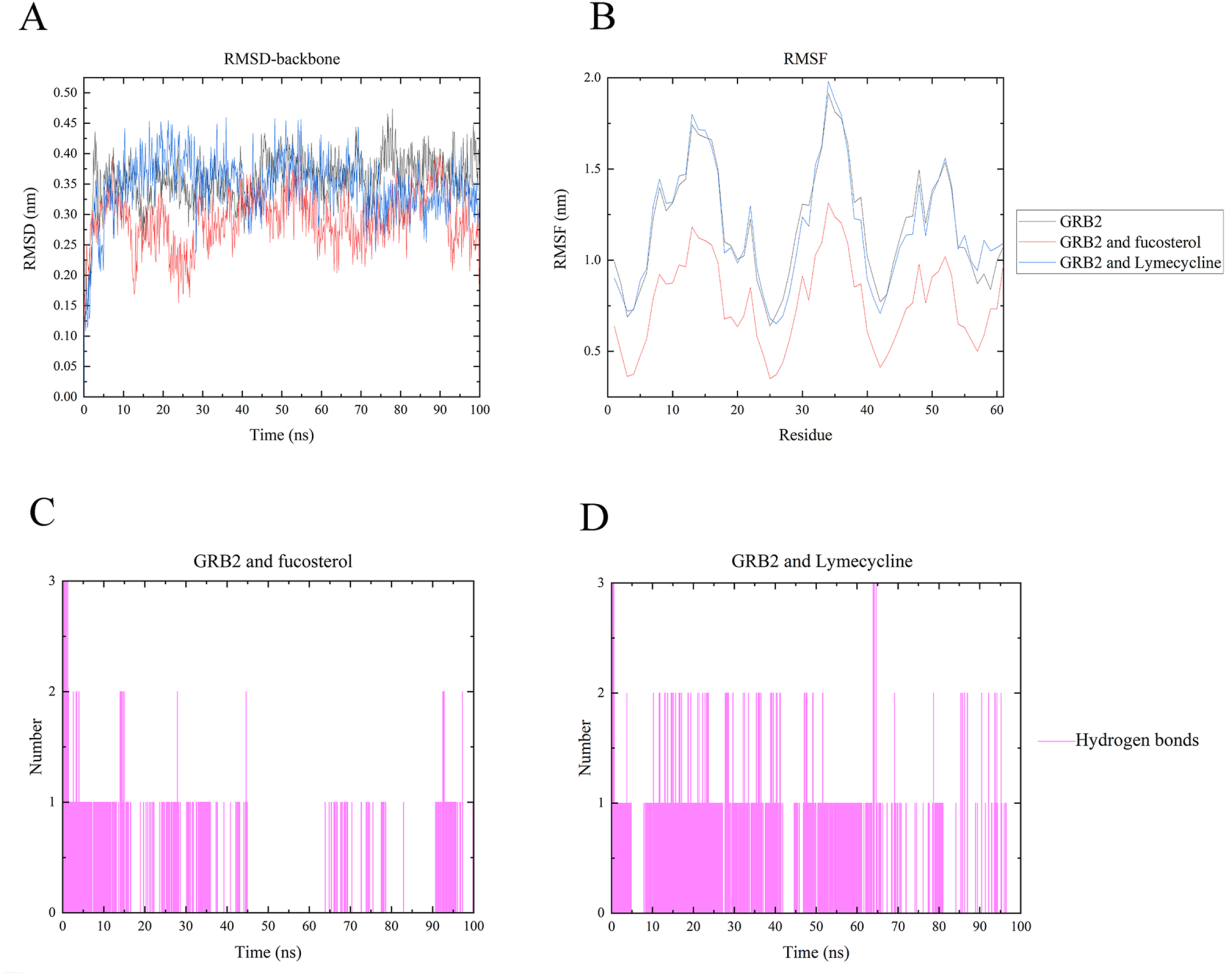
MD simulation interaction diagrams for 100 ns trajectory showing RMSD, RMSF and Hydrogen bond analysis. (**A**) Root mean square deviation (RMSD) of the 100 ns trajectories. (**B**) Root mean square fluctuations (RMSF) of the 100 ns trajectories. (**C**) Hydrogen bond analysis of fucosterol with GRB2. (**D**) Hydrogen bond analysis of Lymecycline with GRB2. The red polyline is shown as GRB2 and fucosterol, the black polyline is shown as GRB2, and the blue polyline is shown as GRB2 and lymecycline. (**A**–**D**) were obtained from Origin2019b (https://www.originlab.com/).

Molecular docking simulation was utilized to verify the binding ability of the fucosterol to the hub genes GRB2, and Lymecycline was selected as the positive control. The docking mode and hydrogen bonding residues of fucosterol and Lymecycline with GRB2 after docking are shown in Fig. 9. Then, the binding sites of ligands, GRB2 and the surrounding residues by software pymol2.4 and ligplot2.3 are shown in Fig. 9A. In the docking model of fucosterol and GRB2, the action site are ASP-16, LYS-11, LYS-21, Ser-33, ASN-30, which forms hydrogen bonds with Ser-33, while the interaction between ligands and surrounding residues is analyzed in Fig. 9B. (binding energy is − 9.9 kcal/mol) It was indicated that the effector-binding energy of fucosterol is mainly provided by hydrophobic interactions rather than hydrogen interactions and it also showed that GRB2 was able to provide a decent hydrophobic environment for the substrate binding. And for positive control Lymecycline, it forms hydrogen bonds with THR-13, LYS-21, ASP-24. The others are hydrophobic interaction (Fig. 9C,D) and the binding energy is − 9.7 kcal/mol. Therefore, the docking effect of fucosterol and GRB2 is not much different from that of Lymecycline, and the binding energy of fucosterol is lower than that of Lymecycline. Figure 10 shows the molecular dynamics of Lymecycline and fucosterol with GRB2 protein ligand complex and GRB2 protein (RMSD, RMSF and hydrogen bond analysis). For RMSD (Fig. 10A), in the 100 ns simulation, all the three systems reached the equilibrium state quickly. In the end, the RMSD value of the complex formed by GRB2 and fucosterol was the lowest (0.25 nm), while the RMSD value of the positive control compound Lymecycline was 0.325 nm, and the RMSD value of the separate GRB2 protein system was 0.4 nm. This suggests that in terms of stability, the fucosterol compound formed a more stable combination in the active pocket of GRB2. For RMSF (Fig. 10B), the GRB2 and fucosterol system showed lowest RMSF, in the range between 0.5 and 1.25 nm, while the other two system between 0.75 and 2.0 nm, which in the docking results fucosterol formed hydrogen bond with SER-33. In RMSF, GRB2 and fucosterol system showed the fluctuations of SER-33 decreased significantly, which means that the hydrogen bonding interaction reduces the flexibility of the system, and Lymecycline formed hydrogen bond with THR-13, LYS-21, ASP-24, the hydrogen bond also reduced the flexibility of this system, but it was not obvious to GRB2 and fucosterol. In terms of hydrogen bond analysis (Fig. 10C,D), the number of hydrogen bonds between fucosterol and GRB2 system was 0 between 45 and 63 ns, while hydrogen bonds were absent in the positive control Lymecycline for a long time. In comparison of the number and existence of hydrogen bonds, the number and duration of hydrogen bonds in Lymecycline and GRB2 system is obviously better than that in fucosterol and GRB2 system. But not only by the hydrogen bonding interaction to determine the combination of ligand and receptor, also other interactions are needed to take into consideration.

### Expression level and immune infiltrates analyses of GRB2 in NSCLC

**Figure 11:**
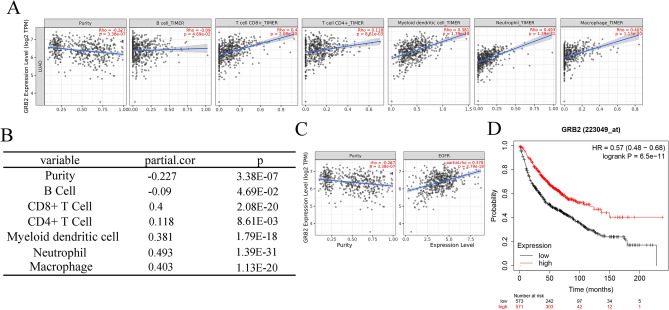
The expression of GRB2 and immune infiltrates in LUAD. (**A**,**B**) Correlation of GRB2 expression with immune infiltration level in LUAD. GBR2 expression had significant positive correlations with infiltrating levels of CD8 + T cells, CD4 + T cells, macrophages, neutrophils and myeloid dendritic cells. (**C**) Relationship between GRB2 and EGFR in LUAD. GRB2 expression showed a strong correlation with EGFR expression after adjusting tumor purity in LUAD. (**D**) Prognostic values of GRB2 (n = 2437) in LUAD (OS in Kaplan–Meier plotter). Line in red with high expression while in black with low expression. Low expression of GRB2 was connected with better over survival.

To further explore the direct relationship between GRB2 and NSCLC, we conducted the analysis of the relationship between GRB2 expression and immune infiltrates on TIMER(2.0) database. As shown in Fig. 11A,B, the expression of GRB2 was significantly negatively associated with tumor purity while significantly positively correlated with immune cells in infiltrating levels, B cells(r = -0.09, *P* = 4.69e−02), CD8 + T cells(r = 0.4, *P* = 2.08e−20), CD4 + T cells (r = 0.118, *P* = 8.61e−03), macrophages(r = 0.403, *P* = 1.13e−20), neutrophils(r = 0.493, *P* = 1.39e−31) and myeloid dendritic cells(r = 0.381, *P* = 1.79e−18), suggesting that the GRB2 expression was mainly related to the immune infiltration of CD8 + T cells, CD4 + T cells, macrophages, neutrophils and myeloid dendritic cells. Additionally, Fig. 11C revealed that GRB2 expression is significantly positively associated with EGFR expression after adjusting tumor purity. What’s more, KM-Plotter analysis performed the result in Fig. 11D that the lower expression level of GRB2 had a better overall survival rate and gene GRB2 was an independent prognosis indictor for OS of patients with LUAD. Hence, we speculated that GRB2 exerted a more significant effect on the prognosis of LUAD, for it was highly associated with various immune cells in LUAD.

## Discussion

NSCLC is characterized by high malignancy, low 5-year survival rate and poor prognosis. However, for patients with advanced lung cancer, having specific predictive biomarkers and receiving targeted therapy or immunotherapy significantly improve quality of life and progression-free survival (PFS) compared to chemotherapy^53–58^. Hence, finding effective targeted drugs to anti-lung cancer has become an urgent problem to be solved. Network pharmacology can analyze and explain the complexity between biological systems, diseases, and drugs from a network perspective, becoming a frontier method of drug discovery^59,60^. Molecular docking is a structure-based drug design method, which can predict the affinity and binding pattern through the interaction between ligands and receptors, accelerate the design and screening of drugs, and provide a basis for future experimental detection^61,62^. Molecular dynamics simulate ligand target complexes in a given system to explore their stability and flexibility^63,64^. Therefore, based on network pharmacology and molecular docking simulation ligand target binding, the mechanism research of fucosterol therapy for NSCLC may serve as the foundation for the development of NSCLC targeted drugs in the future.

Based on the reverse of molecular docking technology for predicting fucosterol targets, and compare with NSCLC related targets, we obtained 37 candidate targets. Fucosterol-NSCLC PPI network analysis uncovered that fucosterol probably exerted pharmacological effects on NSCLC via 37 candidate targets, including 6 hub genes: GRB2, EGFR, MAPK1, SRC, IGF2, MAPK8. According to GO biological process analysis, 37 candidate targets were found to mainly responsible for cell proliferation, apoptosis, migration, signal transduction and angiogenesis. In addition, KEGG analysis of candidate targets disclosed that fucosterol probably has therapeutic effect on NSCLC through multiple pathways like PI3K-Akt signaling pathway, VEGF signaling pathway, ErbB signaling pathway. Furthermore, it is reported that fucosterol has antiproliferative effects on human lung cancer cells by inducing apoptosis, cell cycle arrest and targeting of Raf/MEK/ERK signaling pathway, such as A549 and SK-LU-1 cancer cells, in addition, fucosterol could also inhibit the growth of xenografted tumours in mice^44^. Through the NSCLC-related pathway map, we discover that GRB2 can be acted as the trigger or initiation signal of the Raf/MEK/ERK pathway by PI3K-Akt signaling pathway and ErbB signaling pathway, which may be crucial for fucosterol in the treatment of NSCLC.

Based on molecular docking, the combination of fucosterol and GRB2 can be discussed. At the same time, Lymecycline was selected as the positive control to provide the basis for future experiments. We chose to complete many key structures with higher resolution crystal structures, and then the docking site was selected in the SH3 region of GRB2, so as to influence the cell signal transduction events and achieve the therapeutic effect of cancer. RMSD, RMSF and hydrogen bond analysis were calculated using molecular dynamics simulations to infer the basic properties of ligand-target complexes-stability and flexibility. The stability of RMSD and RMSF was essential to infer good binding affinity, while hydrogen bond analysis was to compare the binding of phytosterol and Lymecycline to GRB2. The results show that the combination of GRB2 and fucosterol is stable.

Growth factor receptor-bound protein 2 (Grb2), a universally expressed adaptor protein, which plays a pivotal downstream mediator role in a variety of oncogenes signaling pathways and has a significant effect on signal transduction in normal and cancer cells^65,66^. Grb2 is a downstream protein of epidermal growth factor receptor (EGFR) that is known to be closely related to NSCLC, and may serve as an adaptor protein to bind to phosphorylated tyrosine in the EGFR, thereby linking receptor activation to intracellular signaling cascade^67–69^. Simultaneously, recent research has demonstrated that the balance of Grb2 monomer–dimer is a determinant of its normal and carcinogenic functions^70^. Grb2 regulating angiogenesis and cell movement is highly overexpressed in tumors and may be useful as a target for anticancer agents. In the present study, we found Grb2 can be targeted by fucosterol and activate the Raf/MEK/ERK pathway to achieve the purpose for treating NSCLC^44,71–73^.

## Conclusion

In summary, our study indicated the molecular and pharmacological mechanism of fucosterol against NSCLC from a systematic perspective. We unveil that GRB2 can serve as an anticancer target in fucosterol to initiate the Raf/MEK/ERK pathway for treating NSCLC. This strategy provides a new idea of anti-NSCLC and lays a foundation for the development of new medicines. Nonetheless, network pharmacology has certain limitations, and more experiments are needed to verify the validity of our findings. Moreover, we hope that our study will be useful for fostering innovative research of marine drugs against cancers.

## Data Availability

The data that support the findings of this study are available from the corresponding author upon reasonable request.

## Abbreviations

NSCLC: Non-small cell lung cancer
LUAD: Lung adenocarcinoma
GRB2: Growth factor receptor-binding protein 2
KEGG: Kyoto encyclopedia of gene and genome
GO: Gene ontology
PPI: Protein-protein interaction
TIMER: Tumor immune estimation resource
